# Productive Performance, Ovarian Follicular Development, Lipid Peroxidation, Antioxidative Status, and Egg Quality in Laying Hens Fed Diets Supplemented with *Salvia officinalis* and *Origanum majorana* Powder Levels

**DOI:** 10.3390/ani11123513

**Published:** 2021-12-09

**Authors:** Ahmed A. Saleh, Shimaa Hamed, Aziza M. Hassan, Khairy Amber, Wael Awad, Mohammed H. Alzawqari, Mustafa Shukry

**Affiliations:** 1 Department of Poultry Production, Faculty of Agriculture, Kafrelsheikh University, Kafrelsheikh 33516, Egypt; sobhikhamis1967@gmail.com (S.H.); khairyamber1957@gmail.com (K.A.); m.alzawqari@gmail.com (M.H.A.); 2 Biology Department of Biotechnology, College of Science, Taif University, P.O. Box 11099, Taif 21944, Saudi Arabia; a.hasn@tu.edu.sa; 3 Animals Production Institute, Agriculture Research Center, Ministry of Agriculture, Giza 12651, Egypt; wawad74@yahoo.com; 4 Department of Animal Production, Faculty of Agriculture and Food Sciences, Ibb University, Ibb 70270, Yemen; 5 Department of Physiology, Faculty of Veterinary Medicine, Kafrelsheikh University, Kafrelsheikh 33516, Egypt; mostafa.ataa@vet.kfs.edu.eg

**Keywords:** *Salvia officinalis*, *Origanum majorana*, performance, antioxidative status, phytoestrogens hormone, laying hens

## Abstract

**Simple Summary:**

Phytoestrogens are plant-derived compounds which can act to mimic estrogen and cause estrogenic effects via binding to estrogen receptors-α and β. Phytoestrogen enhances laying performance during post-peak laying in hens. Hence, the current study investigated the effects of *Salvia officinalis* and/or *Origanum majorana* as phytoestrogen sources on productive performance, ovarian follicular development, lipid peroxidation, antioxidative status, and egg quality in laying hens. A total of 294 (45-week-old Bovans brown hens) were divided into seven experimental groups. The control group was fed with the basal diet; the second and third groups were provided with the same control diet further supplemented with 0.5 and 1 kg/ton *Salvia officinalis*, respectively; the fourth and fifth groups received the control diet further supplemented with 0.5 and 1 kg/ton *Origanum majorana*, respectively; while the sixth group was offered a diet supplemented with 0.5 kg/ton *Salvia officinalis* and 0.5 kg/ton *Origanum majorana*. Finally, the seventh group received a diet supplemented with 1 kg/ton *Salvia officinalis* and 1 kg/ton *Origanum majorana*. The results reveal that dietary supplementation with *Salvia officinalis* and/or Origanum improved productive performance, ovarian follicular development, antioxidant activity, hormonal status, and steroidogenesis in Bovans brown laying hens.

**Abstract:**

This study aimed to assess the effect of dietary supplementation with different levels of *Salvia officinalis* and/or *Origanum majorana* on productive performance, ovarian follicular development, lipid peroxidation, antioxidative status, and egg quality in laying hens. Two hundred and ninety-four 45-week-old Bovans brown hens were allocated into seven groups, with seven replicates of six hens each. The first group was fed with the basal considered as a control (A); the second (B) and third (C) groups were provided with the same control diet further supplemented with 0.5 and 1 kg/ton *Salvia officinalis*, respectively; the fourth (D) and fifth (E) groups received the control diet further supplemented with 0.5 and 1 kg/ton *Origanum majorana*, respectively; while the sixth (F) and the seventh (G) groups were offered a diet supplemented with 0.5 kg/ton *Salvia officinalis* and 0.5 kg/ton *Origanum majorana* and 1 kg/ton *Salvia officinalis* and 1 kg/ton *Origanum majorana*, respectively. No significant effects were observed in the final body weight (BW) and feed intake (FI) of the laying hens. In the diets supplemented with *Salvia officinalis* and *Origanum majorana*, the egg weights for groups C, F, and G had significantly higher values only compared to group D. The supplementation of the diets with *Salvia officinalis* and/or *Origanum majorana* significantly (*p* < 0.05) increased the Follicle stimulating hormone (FSH), luteinizing hormone (LH), and estradiol estrogenic hormone concentration, except for *Origanum majorana* at both levels with regard to estradiol. The dietary utilization of *Salvia officinalis* and *Origanum majorana* did not significantly alter the plasma glutamic oxaloacetic transaminase (GOT) and glutamic pyruvic transaminase (GPT), total protein, albumin, globulin, and High density lipoprotein (HDL) parameters. Cholesterol, glucose, triglyceride, and Low density lipoprotein (LDL) were decreased (*p* < 0.05) in the birds fed with *Salvia officinalis* and/or *Origanum majorana* supplemented diets. Moreover, at both doses, the dietary supplementation with *Salvia officinalis* and *Origanum majorana* decreased (*p* < 0.05) the yolk cholesterol and liver Malondialdehyde (MDA) levels. In addition, the dietary enrichment with *Salvia officinalis* and/or *Origanum majorana* decreased (*p* < 0.05) the palmitoleic and stearic fatty acids’ egg yolk concentration. In contrast, the yolk linoleic fatty acid concentration was significantly increased by *Salvia officinalis* and/or *Origanum majorana*. In conclusion, dietary supplementation with *Salvia officinalis* and/or Origanum positively affected productive performance, ovarian follicular development, antioxidant activity, hormonal status, and steroidogenesis in Bovans brown laying hens.

## 1. Introduction

Nowadays, one of the top priorities in poultry production is identifying new feed supplementation alternatives that will boost animal health and quantitative and qualitative production parameters [1]. Traditional medicine already uses some of these plants as a natural source of antioxidants to control and cure several diseases [2]. Many medicinal plants have various biological properties due to the abundance of active components which improve animal growth and immunity [3,4].

Additionally, medicinal plants are used as natural feed additives in poultry diets [5]. Using herbs or extracts is a fast and easy way of distributing natural antioxidants into their bodies [6]. Antioxidants are involved in preserving animal health, assisting the immune system, and enhancing the animal’s productive efficiency [7]. At the same time, herbal additives are less harmful, have fewer side effects than synthetic products, and can be incorporated into feed or water to combat poultry diseases [8].

Phytoestrogens are herbal compounds that imitate estrogen and induce estrogenic effects by binding to estrogen receptors [9]. Dietary daidzein, a natural phytoestrogen, improved Shaoxing duck laying efficiency during the post-peak laying period [10]. Dusza et al. showed that supplementation with daidzein improved egg development and eggshell thickness while lowering the broken eggs rate [9]. Furthermore, Saleh et al. noted that improved production, egg quality, antioxidative status, hormonal profile, and steroidogenesis were observed with the dietary application of mixed phytoestrogen sources (flaxseeds and fenugreek seeds) [11].

The sage plant, *Salvia officinalis*, could be contained in these herbal additives. It is the most common and oldest plant medicine used in ancient and modern medicine. It includes volatile oils, flavonoids, and phenolic acids, with multifaceted beneficial properties [12,13].

Moreover, these aromatic plants of the Labiatae family have been used in poultry and animals feed due to their antimicrobial and antioxidant activity [9]. The dietary supplementation of *Salvia officinalis* reduced the Salmonella counts in the liver, spleen, and cecum of broilers infected with Salmonella enteritidis [14]. Additionally, *Salvia officinalis* L. extract supplementation in the broilers’ diet significantly improved their body weight gain and other growth performance parameters, whereas in another experiment, Ryzner et al. [15] showed that *Salvia officinalis* reduced the oxidative stress parameters. Recently, the supplementation of *Salvia officinalis* in broilers’ diets improved the immunity response of broilers and significantly decreased the ileal counts of *E. coli* [16]. Elsewhere, with *Salvia officinalis* supplementation in laying hen diets, antioxidant defense mechanisms were improved by the induction of antioxidant enzymes [17].

Oregano (Origanum vulgare) belongs to the Lamiaceae family. Carvacrol and thymol are its main active compounds, accounting for 78–82% of the total oil [18].

When used in the poultry diets, oregano may exert antioxidant properties since it has two important phenol compounds corresponding to 78–85% of the oil’s composition, namely carvacrol (2-methyl-5-isopropyiphenol) and thymol (2-isopropyl-5-methylphenol) (Basmacioglu Malayoğlu et al., 2010) [19]. According to Botsoglou et al., these compounds possess antimicrobial activity [20] acting to reduce undesirable intestinal microflora which favors the absorption of nutrients [21]. The essential oils content in oregano can act as stimulant agents of the immune system during acute or chronic inflammatory processes that can be characterized by an increase in the levels of serum globulins [22], which can express the metabolic and nutritional status of poultry [23]. Moreover, essential oils may improve nutrient digestion and absorption by enzymatic stimulation and they also may exert positive effects when used in laying hens.

Hussain et al. [24] found that *Salvia officinalis* essential oil possesses excellent radical scavenging activity. Similarly, oregano essential oil’s dietary supplementation increased the egg production rate, eggs’ weight, and hens’ FCR. These findings may be attributed to oregano essential oil’s higher biological activity when used in laying hens’ diets [25]. Mathlouthi et al. indicated that dietary supplementation with the essential oils of thyme, sage, and rosemary substantially increased FCR (*p* < 0.05) [26]. The usage of 1.0% oregano in the diet improved egg production and egg weight in hens according to Radwan et al. [27]. Moreover, these medicinal herbs are rich in flavonoids, improving the immune system and generating antibodies [28].

Considering the available literature, the evidence for the use of *Salvia officinalis* L. and Origanum vulgare and their mixture in the diets of organic laying hens is limited. Hence, the objective of the present study was to assess the impact of dietary supplementation with *Salvia officinalis* and/or *Origanum majorana* on the production efficiency, ovarian follicular development, lipid peroxidation, antioxidant status, and egg quality traits in the Bovans brown laying hens

## 2. Materials and Methods

### 2.1. Ethical Approval

This work was performed following recommendations from the Local Experimental Animal Care Committee on Ethics, University of Kafrelsheikh, Egypt (Number 4/2016EC).

### 2.2. Total Polyphenols Content in Salvia officinalis and Origanum majorana Powder Levels

The polyphenol content was determined by the method described by Slinkard and Singleton [18] with some modifications (Table 1). Before the analysis, the samples of extracts were diluted (1:20). The findings were determined as equivalents of gallic acid (G.A.) and expressed as mg/L. The measurements were implemented in triplicate using the Japan spectrophotometer JASCO V-530–MEDSON (Tokyo, Japan).

### 2.3. Birds and Experimental Diets

In an open-sided shed, two hundred and ninety-four 45-week-old Bovans brown hens underwent 25 weeks of production, with an egg production rate of 75%, kept individually in laying cages under 16 h: 8 h light/dark cycle under summer conditions and the same management protocol (temperature, moisture, ventilation). Birds were randomly allotted into seven groups (with seven replicates of 6 hens per replicate and each bird housed individually). The cage was a Big Dutchman with regular dimensions of 40 × 35 × 60 cm^3^ and was a double-sided battery cage. An automated nipple drinker was given in each cage. The first group was fed the basal unsupplemented diet (control, A); the second (B) and third (C) groups were fed the basal diet further supplemented with 0.5 and 1 kg/ton *Salvia officinalis*, respectively; the fourth (D) and fifth (E) groups received the basal diet supplemented with 0.5 and 1 kg/ton *Origanum majorana*, respectively; the sixth (F) group was fed the basal diet further supplemented with 0.5 kg/ton *Salvia officinalis* and 0.5 kg/ton *Origanum majorana*. Finally, the seventh (G) group was fed the basal diet supplemented with 1 kg/ton *Salvia officinalis* and 1 kg/ton *Origanum majorana*. The constituents of the basal diet, which was prepared to comply with the recommendations of [29], are presented in Table 2. No any mortality occurred during the experiment.

### 2.4. Laying Performance

At 45 and 57 weeks of age, the birds were weighed while the feed intake was recorded weekly and expressed on a cage basis. Eggs were collected and weighed daily, and the average of their values during the experimental period was used to assess the egg production (% hens–day) and egg weight. Egg mass was estimated by multiplying the egg production with egg weight. The overall feed conversion ratio was calculated on a cage basis as g feed/g egg.

### 2.5. Egg Quality

The assessment of the egg and shell feature parameters, namely the egg weight, yolk color, shell thickening, yolk width, white width, yolk height, egg white high, yolk weight, egg white weight, and shell weight was performed at 45 and 57 weeks. For this purpose, 70 eggs/group laid between 08:00 and 12:00 h were arbitrarily selected, weighed, and prepared for evaluation. Yolk color was assessed using the Roche yolk color fan method [30]. Shell weight was calculated according to the following method; egg coats were washed from any adherent albumen, then dried and weighed, proportional to the whole egg weight. The eggs’ regularity was evaluated on individual eggs, corresponding to the measured egg weight.

### 2.6. Reproductive Morphological Assessment

At the end of the experiment (57 weeks of age), ten birds were randomly selected from each group and weighed, anesthetized, and euthanized by decapitation. The reproductive system weight and morphology were also recorded. Liver, gizzard, spleen, and abdominal fat weight were also recorded. The ovary and oviduct were also assessed by measuring the oviduct’s total length. Oviduct parts were assessed and their relative weight and length were assessed for oviduct parts (including vagina, uterus, isthmus, magnum, and infundibulum). Liver samples were kept at −24 °C for consequent analysis. The ovary and oviduct were separated and weighed. The weight and number of large yellow follicles (LYFs) (10 mm diameter), small yellow follicles (SYFs) (5–10 mm diameter), large white follicles (LWFs) (3–5 mm diameter), and medium white follicles (MWFs) (1–3 mm diameter) were estimated as described by Slinkard et al. [31]. The stroma weight includes the remaining ovarian tissue after counting and the removal of the LYFs.

### 2.7. Blood Examination

At the end of the experiment, blood samples were obtained in heparinized tubes from the wing vein and centrifuged at 3000× *g* for 20 to obtain plasma samples, kept at −20 °C until analysis. The plasma concentrations of the total lipid, total cholesterol, triglyceride, and high and low-density lipoproteins (HDL and LDL), total protein, albumin, and glucose levels were analyzed using a colorimetric kit (Egyptian Company for Biotechnology, Cairo, Egypt, and Wako Chemicals, VA, USA). The content of liver malondialdehyde (MDA) was determined utilizing a commercial colorimetric kit (Liquizyme MDA; Biotechnology, Egypt). The absorbance was monitored using a spectrophotometer (Unico UV 2000; SpectraLab Scientific Inc., Alexandria, VA, USA) at a wavelength of 545 nm. The plasma luteinizing hormone (L.H.), follicle-stimulating hormone (FSH), and estradiol 17β were quantified utilizing a homologous RIA [32].

### 2.8. Egg Yolk Fatty Acids Profile

At 57 weeks of age, 70 eggs were collected per group to measure the yolk fatty acid profile in egg yolk, including linolenic, oleic, palmitic acid, total cholesterol, vitamin E, and calcium concentration. A Shimadzu GC-4 CM gas chromatograph (PFE), outfitted with a flame ionization detector (FID), was used. A normal methyl ester mixture was prepared before the samples were measured under similar conditions. The retention times of the unknown methyl ester samples were compared to that of the standard. The concentrations of methyl esters were determined as previously described (Radwan, Saleh et al.) by [19,28]. The HPLC determined vitamin E and the total concentration of cholesterol in egg yolk using the technique described by [28].

### 2.9. Data Analysis

The differences between the treatment groups and the control group were analyzed with a general liner model using SPSS (Version 17.0). One-way ANOVA was applied to determine the effects of *Salvia officinalis* and/or *Origanum majorana*, when birds were the statistical units for performance parameters, organs’ weights and samples for biochemical and other parameters. Duncan’s new multiple range tests were used to identify which treatment conditions were significantly different from each other at a significance level of *p* < 0.05.

## 3. Results

### 3.1. Laying Performance

The effect of dietary *Salvia officinalis* and *Origanum majorana* treatments on final body weight (BW), body weight gain (BWG), F.I., and egg weight in laying hens during the experimental period are presented in Table 3. No significant effects were detected in the final BW, BWG, and F.I. of laying hens fed dietary supplemented diets with different quantities of *Salvia officinalis* and *Origanum majorana* compared to the control. On the other hand, the egg weight, egg production, and egg mass were significantly improved by a combined 1.0 kg/ton *Salvia officinalis* with 1.0 kg/ton *Origanum majorana* in laying hens. The dietary supplementation with 1.0 kg/ton of each *Salvia officinalis* and *Origanum majorana* recorded the most significant improvement in FCR value compared (*p* < 0.05) to the control. The dietary supplementation of both powders with 0.5 kg/ton also increased the FCR.

### 3.2. Egg Quality

The data on egg quality from 45 to 57 weeks of age in laying hens are illustrated in Table 4. At the initial period of the study (45 weeks), no significant variations concerning egg quality characteristics were recorded. Dietary supplementation with 1.0 kg/ton of *Salvia officinalis* or/and 1.0 kg/ton of *Origanum majorana* increased egg yolk color at 57 weeks. Moreover, the supplementation of 1.0 kg/ton of *Salvia officinalis* with 1.0 kg/ton of *Origanum majorana* also improved the control group’s shell thickness (*p* < 0.05). However, the dietary supplement of 0.5 kg/ton of *Salvia officinalis* combined with a 0.5 kg/ton of *Origanum majorana* increased the egg white width (*p* < 0.05) compared to the other bird groups. *Salvia officinalis* and/or *Origanum majorana* dietary supplementation did not alter the egg weight, yolk width, yolk height, egg white height, yolk weight, egg white weight, and shell weight in laying hens during the experimental period.

### 3.3. Reproductive Morphology Measurements

The results regarding the effects of dietary *Salvia officinalis* and *Origanum majorana* treatments on internal organs weight and reproductive morphology are shown in Table 5. Supplementation with *Salvia officinalis* and *Origanum majorana* did not affect the relative organs’ weights (gizzard, liver, spleen, heart and abdominal fat) and the weight of the ovary, oviduct, vagina, uterus, isthmus, magnum, and infundibulum. However, the diets supplemented with 1 kg of *Salvia officinalis* or 1 kg of *Origanum majorana* or combining *Salvia officinalis* with *Origanum majorana* increased the oviduct length and uterus length (*p* < 0.05) compared to the control group. Birds fed 0.5 kg *Salvia officinalis*/ton had the highest large yellow follicle number. In comparison, those fed 0.5 kg *Origanum majorana*/ton had the highest number of medium white follicles compared to other groups. However, the large white follicle value was the highest (*p* < 0.063) in the *Salvia officinalis* group at a 1.0 kg/ton level.

### 3.4. Blood Constituents

As shown in Table 6, the dietary utilization of *Salvia officinalis* and *Origanum majorana* did not significantly alter the plasma GPT, GOT, total protein, albumin, globulin, and HDL parameters. Cholesterol and triglyceride were significantly decreased by *Origanum majorana* groups and the mixture of *Origanum majorana* and *Salvia officinalis* groups compared with the control and *Salvia officinalis* groups while plasma LDL was decreased (*p* < 0.05) in birds fed *Salvia officinalis* and *Origanum majorana* mixture supplemented diets compared with others groups, however, the lowest glucose optioned by C and G groups compared with other groups.

**Figure 1:**
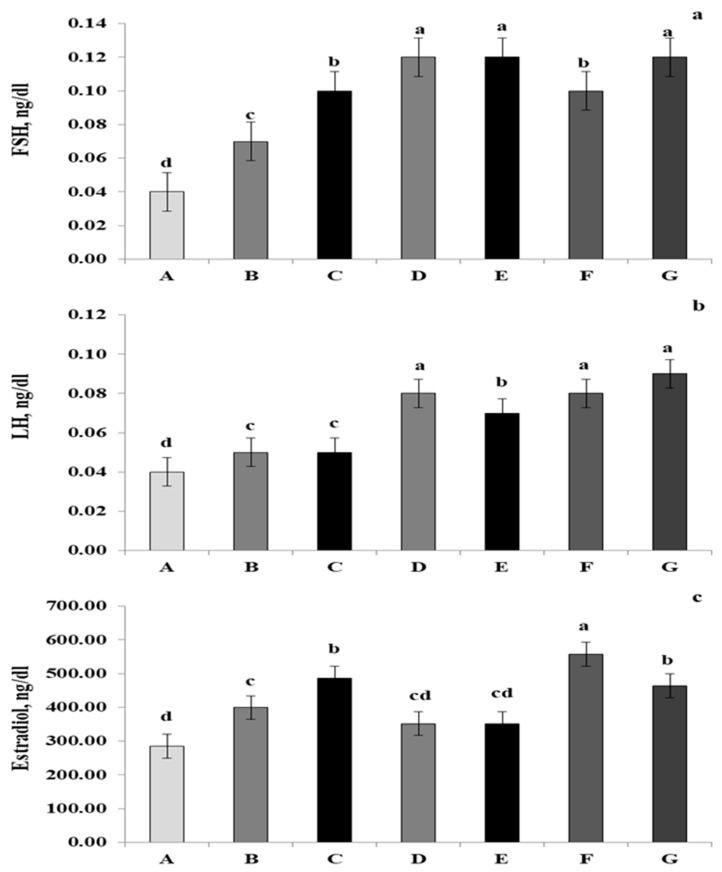
The effects of dietary *Salvia officinalis* and *Origanum majorana* treatments on FSH (**a**), L.H. (**b**), and estradiol (**c**). a, b, c, d: The mean values of the different letters in the same row are significantly different (*p* < 0.05). The values presented are means and their standard error of 60 per treatment. Abbreviations: control (A); a basal diet supplemented with 0.5 *Salvia officinalis* (B); a basal diet supplemented with 1 kg/ton *Salvia officinalis* (C); basal diet supplemented with 0.5 *Origanum majorana* (D); a basal diet supplemented with 1 kg/ton *Origanum majorana* (E); a basal diet supplemented with 0.5 kg/ton *Salvia officinalis* and 0.5 kg/ton *Origanum majorana* (F); a basal diet supplemented with 1 kg/ton *Salvia officinalis* and 1 kg/ton *Origanum majorana* (G).

As shown in Figure 1a–c, the FSH, L.H. and estrogenic hormones significantly increased (*p* < 0.05) as an effect of the supplementation with 1.0 kg/ton of *Salvia officinalis* and/or *Origanum majorana*. Simultaneously, the combined supplementation of 0.5 kg/ton *Salvia officinalis* and 0.5 kg/ton *Origanum majorana* increased the estradiol levels compared to all other groups.

### 3.5. Yolk Chemical Profile

**Figure 2:**
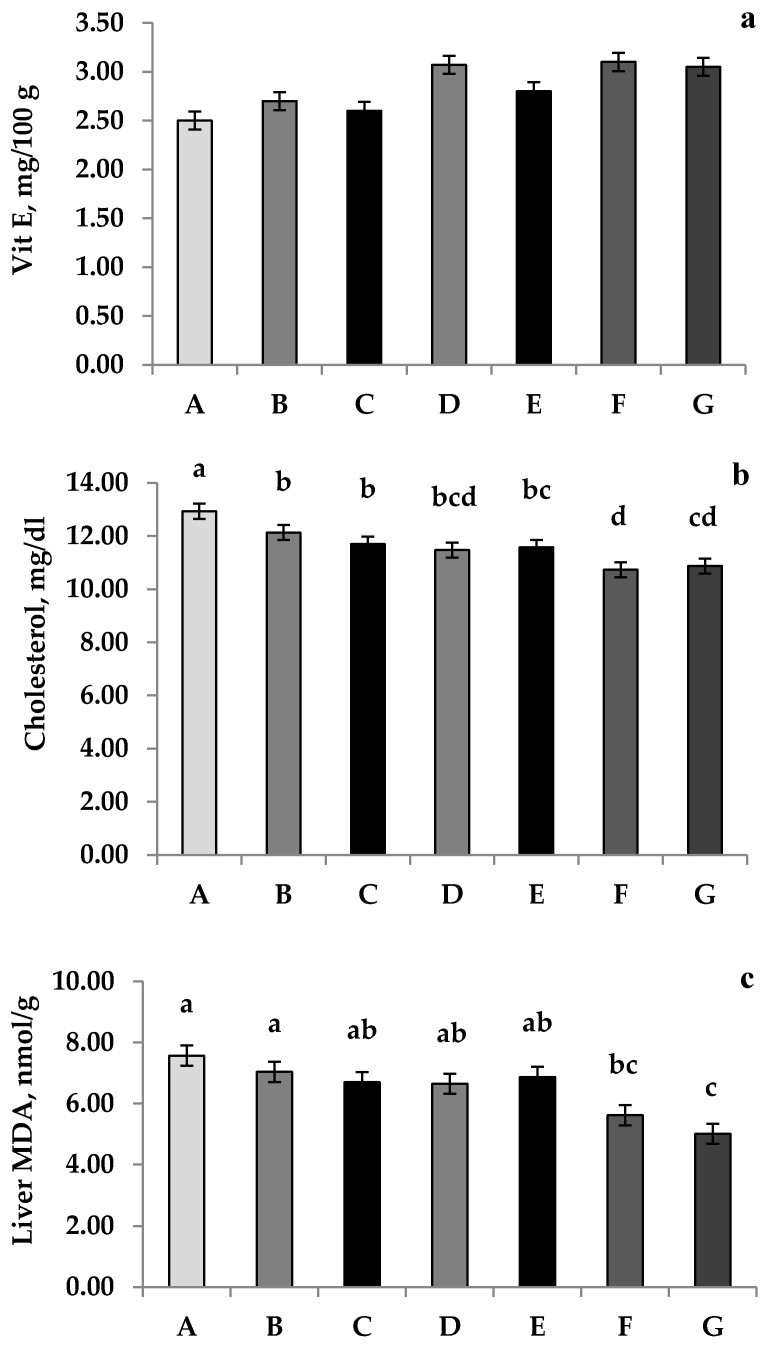
The effects of dietary *Salvia officinalis* and *Origanum majorana* treatments on Vit E (**a**), cholesterol (**b**), and Liver MDA (**c**). a, b, c, d: The mean values followed by the different letters in the same row are significantly different (*p* < 0.05). The values presented are means and their standard error of 60 per treatment. Abbreviations: control (A); basal diet supplemented with 0.5 *Salvia officinalis* (B); basal diet supplemented with 1 kg/ton *Salvia officinalis* (C); basal diet supplemented with 0.5 *Origanum majorana* (D); basal diet supplemented with 1 kg/ton *Origanum majorana* (E); basal diet supplemented with 0.5 kg/ton *Salvia officinalis* and 0.5 kg/ton *Origanum majorana* (F); basal diet supplemented with 1 kg/ton *Salvia officinalis* and 1 kg/ton *Origanum majorana* (G).

The effects of *Salvia officinalis* and/or *Origanum majorana* dietary supplementation on the yolk fatty acid profile analysis in laying hens are shown in Table 7; the dietary utilization of *Salvia officinalis* and/Majorana decreased (*p* < 0.05) the concentrations of palmitoleic and stearic fatty acids in egg yolk. In contrast, the yolk linoleic fatty acid concentration was significantly increased compared with the control group. However, no substantial variations were detected in the other yolk fatty acid contents. The dietary supplementation of *Salvia officinalis* and *Origanum majorana* significantly decreased (*p* < 0.05) the egg yolk cholesterol compared to the control group; however, no significant effect was observed on the Vit. E yolk content (Figure 2a,b). The lipid peroxidation index (are MDA) levels presented in Figure 2c; it could be observed that the combination of *Salvia officinalis* and *Origanum majorana* reduced (*p* < 0.05) liver MDA concentrations in laying hens.

## 4. Discussion

There are limited data on the effect of *Salvia officinalis* and *Origanum majorana* powder and their combined effects in laying hens. The present study showed that dietary supplementation with *Salvia officinalis* and *Origanum majorana* powder at a level of 1 kg/ton diet significantly increases egg production, egg weight, egg mass, and improves FCR at 57 weeks of age (*p* < 0.05, Table 3). Consistent and contradictory results were documented regarding the supplementation of *Salvia officinalis* and *Origanum majorana* and their potential effects on the laying hens’ productive performance. In a study by Özek [33], the supplementation of the laying hens’ diets with essential oils, including *Salvia officinalis* and *Origanum majorana*, did not affect the product performance, apart from in terms of the egg weight and FCR, which was meaningfully higher in the treated groups than in the control group. Moreover, Bölükbası et al. [34] showed that the inclusion of a 200 mg/kg extract of *Salvia sclarea* L. reduced the feed consumption of laying hens. In the latter study, the feed conversion was also improved in the hens supplemented with *Salvia sclarea* L., but there was no effect on the body weight or laying performance. Moreover, the supplementation of *Salvia officinalis* L. leaves at 2.5% in the laying hens’ diet did not improve any of the performance parameters [35]. The supplementation of the extract of Salvia sclarea L. in the diets of laying hens did not affect the body weight or laying percentage but improved the feed conversion ratio [36]. Several researchers have found results which are consistent with our findings [37,38], having discovered that supplementing broiler breeder and brown laying hen diets with mixed herbs essential oils, including those of *Salvia officinalis* and *Origanum majorana*, significantly increased egg production. On the other hand, supplementation did not affect the broiler breeder egg development, egg weight, or egg production at 30–40 weeks of age in other studies. These discrepancies could possibly be attributed to the dose, nature, and source of plant extracts added to the diet; additionally, adding these herbs’ powders to the diet may improve gut health and feed digestibility, thereby enhancing the performance of the laying hens [39].

As presented in Table 4, supplementing the laying hens’ diets with a high level of *Salvia officinalis* with or without *Origanum majorana* substantially improved yolk color and shell thickness. When the diets were supplemented with rosemary, oregano, or saffron, changes in yolk color were also recorded by Surai and Sparks [40]. The yolk’s yellow color is linked to the amount of xanthophyll in the diet and the antioxidant activity of pigments such as carotene and xanthophyll that protect lipids from oxidation [41,42]. Alagawany et al. [7] reported that it is well known and well established that the color of the yolk is certainly and significantly related to the carotenoid content. However, depending on the form of carotenoids present and the molecules’ chemical structure, the effectiveness of carotenoids’ accumulation in egg yolk and their effect on yolk coloration greatly varies. Since carotene is almost entirely converted into vitamin A or otherwise metabolized, chicken is characterized by an almost exclusive accumulation of xanthophylls, the main contributor to chicken egg yolk pigmentation [40]. Saleh et al. [43] found that eggshell thickness was markedly enhanced with herbal additives and essential oils. The serum concentration of calcium associated with phytoestrogens was increased, which is most likely responsible for the eggshell characteristics observed in this study [44,45].

In our experiment, the improved egg quality could be attributed to the higher abundance of antioxidant substrates in a specific group which helped the birds better tolerate thermal stress during the production of the yolk [46]. The improved digestion and absorption of nutrients in the groups supplemented with *Salvia officinalis* L. could also have contributed to the higher yolk weight [47,48]. Similarly, Bozkurt et al. [49] reported that the yolk weight was improved when the diets of the laying hens were supplemented with *Salvia triloba* L. extract. In others studies, the hens were subjected to thermal stress conditions, and the outcome was an increase in yolk weight with a concurrent reduction in albumen weight. In the past, others have also investigated the effects of *Salvia officinalis* L. supplementation on egg quality characteristics. Loetscher et al. [50] showed that the dietary supplementation of leaves of *Salvia officinalis* L. at 2.5% did not improve the egg quality parameters. On the other hand, the supplementation of an extract of Salvia sclarea L. increased the egg weight and Haugh units, while it reduced the yolk percentage [51].

As revealed in Table 5, dietary supplementation with *Salvia officinalis* and *Origanum majorana* powder decreased the liver weight and isthmus weights. The effects of *Salvia officinalis* and *Origanum majorana* on oviduct and ovarian morphology have never been studied before. The essential phytoestrogens present in natural plants are usually isoflavones [52]. Some herbs used in feed additive blends have estrogenic effects which may affect oviduct development. The flavonoids present in marigold and fennel seeds help prevent menopausal symptoms in laying hens [53]. In contrast, the herbal feed supplement increased ovarian weight and small follicle count [54]. Therefore, in the current study, the higher value of large yellow follicles was obtained by supplementation with *Salvia officinalis*. Still, the higher medium white follicles were obtained in the treatment supplemented with 0.5 kg/ton of *Origanum majorana*. At the same time, the ovary, oviduct, vagina, uterus, and isthmus weights and infundibulum length were not influenced by treatment groups. As shown in Table 6, dietary supplementation with *Salvia officinalis* and *Origanum majorana* significantly decreased the glucose, total cholesterol, and LDL concentration in the blood plasma of laying hens (*p* < 0.05). Previous research has shown that using oregano oil in the broiler diet reduces poultry’s cholesterol content [55]. Additionally, Ebeid et al. revealed that the serum triglyceride and total cholesterol levels were considerably depressed by rosemary or oregano dietary supplementation in laying hens. The effect of *Salvia officinalis* with/or *Origanum majorana* on lipid digestion may explain cholesterol reduction, as shown in our results [56].

Similarly to the results observed in this study, Bampidis et al. observed the absence of differences regarding the cholesterol levels in turkeys fed dried oregano leaves [57]. Moreover, diets used for broilers containing carvacrol and thymol (Lee et al., 2003b) and used for laying hens with a mixture of essential oils [58] caused no differences on cholesterols levels. This non-significant effect may be associated with oregano essential oil components that were ineffective in the inhibition of the enzyme 3-hidroxi-3-methyl-glutaril198 CoA reductase (HMG-CoA reductase) which serves to limit cholesterol synthesis [59].

Hashemipour et al. discovered that feeding poultry with herbs or their constituents (thymol and carvacrol) increased serum antioxidant enzyme activities, such as SOD and glutathione peroxidase, thus lowering the MDA levels properties of the eggs but also protecting laying hens from pro-oxidative conditions [60]. Loetscher et al. [61], showed that the supplementation of leaves of *Salvia officinalis* L. at a level of 2.5% improved the antioxidative properties of egg yolks. Elsewhere, it was shown that the supplementation of an extract also containing *Salvia triloba* L. resulted in a significant reduction in MDA in egg yolks and increased the levels of liver enzymes involved in the antioxidative pathways [62,63].

Our findings in Figure 1 are consistent with those of [11,64], who found that adding phytoestrogen to the diets of laying hens may increase steroidogenesis and laying rate. They also found that flaxseeds, fenugreek seeds, and their combination significantly affected LH and FSH plasma concentrations. It can be inferred from the current findings on the effects of *Salvia officinalis* and *Origanum majorana* supplementation as phytoestrogens that it had an impact on the hormone status and thus positively affected the egg production rate, egg quality, and other chemical properties of eggs. As presented in Table 7, the dietary utilization of *Salvia officinalis* and *Origanum majorana* significantly increased the α-linolenic acid content in egg yolk. However, palmitoleic and stearic saturated fatty acids were decreased. Simopoulos and Saleh et al., 2021 [65,66] showed that unsaturated fatty acids are essential in animal and human nutrition as they help prevent diseases such as coronary artery disease, hypertension, and diabetes. It is well established that *Salvia officinalis* is a wealthy supplier of fatty acids [11]. Likewise, unsaturated fatty acids increased whilst saturated fatty acids reduced in egg yolks when chickens were fed diets have flaxseed, red pepper, and fenugreek seeds, according to [67].

## 5. Conclusions

In laying hens, phytoestrogens can elicit biological responses similar to those produced by endogenous estrogen. Supplements containing phytoestrogens such as *Salvia officinalis* and *Origanum majorana* may be used in laying hen diets, with prospective rewards for laying hens’ efficiency, egg quality, antioxidative status, hormonal profile, and steroidogenesis. In conclusion, this study found that supplementing Bovans brown laying hens with a combination of 1 kg/ton *Salvia officinalis* and 1 kg/ton of *Origanum majorana* improved their productive performance (egg weight, egg production, and FCR); in addition, blood glucose and total cholesterol were decreased. Consequently, these findings may highlight the value of combined *Salvia officinalis* and *Origanum majorana* supplementation in the diets of laying hens.

## Figures and Tables

**Table 1:** Phenolic compounds’ content in *Salvia officinalis* and *Origanum majorana*.

Compound	*Salvia officinalis*	*Origanum majorana*
Total phenolic content, (mg GAE/g DW) ^1^	26.07	34.37
Sinapinic acid, μg/g DW	0.787	2.247
*P*-coumaric acid, μg/g DW	0.711	1.734
Ferulic acid, μg/g DW	9.236	8.369
Hesperidin, μg/g DW	3.601	4.967
Isorhamnetin, μg/g DW	0.595	2.572
Catechin, μg/g DW	0.537	1.546
Rutin, μg/g DW	10.131	11.193
Quercetin, μg/g DW	0.228	1.209

^1^ Data expressed as mg of gallic acid equivalents per g dry weight (D.W.).

**Table 2:** Composition and nutrient levels of the basal diet.

Ingredient	g/kg
Corn	603
Soybean meal, 46%	228
Gluten meal, 62%	45
Soybean oil	17
Dicalcium phosphate	20
DL-methionine, 99%	2.1
Threonine, 99%	0.5
Limestone	72
NaCl	3
Vitamin mineral premix ^1^	4
Sodium bicarbonate	2.4
Potassium carbonate	3
Total	1000
**Calculated nutrient levels ^2^**	
Crude protein, %	18.06
ME, Kcal/kg diet	2854
Calcium, %	3.27
Total phosphorus, %	0.72
Available phosphorus, %	0.47
Ether extract, %	4.44
Fiber, %	2.80
Lysine, %	0.88
Methionine, %	0.49
**Chemical analysis, %**	
Crude protein, %	18.02
Ether extract, %	4.42
Calcium, %	3.25
Total phosphorus, %	0.70

^1^ Vitamin mineral premix (units per kilogram of feed): vitamin A, 10,000 IU; vitamin D3, 3500 IU; vitamin E, 35 IU; menadione, 1.5 mg; riboflavin, 5 mg; pantothenic acid, 8 mg; vitamin B12, 0.012 mg; pyridoxine, 1.5 mg; thiamine, 1.5 mg; folic acid, 0.5 mg; niacin, 30 mg; biotin, 0.06 mg; iodine, 0.8 mg; copper, 10 mg; iron, 80 mg; selenium, 0.3 mg; manganese, 80 mg; zinc, 80 mg; ^2^ Calculated according to NRC (1994) for brown Bovens laying hens.

**Table 3:** Effect of different treatments on the productive performance in laying hens.

Item	Treatments Group							SEM	*p*-Tukey
	A	B	C	D	E	F	G		
Initial body weight, g	1933.89	1942.78	1946.11	1944.44	1945.56	1951.11	1953.17	14.54	0.982
Final body weight, g	2025.00	2023.89	2018.33	2032.78	2034.44	2035.83	2039.11	24.17	0.917
Body weight gain, g	91.11	81.11	72.22	88.33	88.89	84.72	85.94	26.16	0.899
Feed intake g/d	116.2	116.8	115.2	115.7	115.9	114.9	116.1	9.14	0.762
Egg weight, g	62.49 ^ab^	62.03 ^ab^	63.15 ^a^	60.50 ^b^	62.54 ^ab^	63.16 ^a^	63.31 ^a^	0.50	0.019
Egg production, %	71.36 ^bc^	68.98 ^c^	76.99 ^a^	71.76 ^bc^	74.14 ^ab^	75.20 ^ab^	78.17 ^a^	1.04	0.000
Egg mass, g of egg/hen/d	44.47 ^bc^	42.73 ^c^	48.63 ^ab^	43.43 ^c^	46.40 ^ab^	47.49 ^ab^	49.49 ^a^	1.00	0.002
FCR, g feed/g egg	2.61 ^ab^	2.72 ^a^	2.37 ^b^	2.66 ^ab^	2.49 ^b^	2.42 ^b^	2.34 ^b^	0.26	0.032

^a,b,c^ The mean values of the different letters in the same row are significantly different (*p* < 0.05). The values presented are the means and their standard error of 60 per treatment. Abbreviations: control (A); a basal diet supplemented with 0.5 *Salvia officinalis* (B); a basal diet supplemented with 1 kg/ton *Salvia officinalis* (C); basal diet supplemented with 0.5 *Origanum majorana* (D); a basal diet supplemented with 1 kg/ton *Origanum majorana* (E); a basal diet supplemented with 0.5 kg/ton *Salvia officinalis* and 0.5 kg/ton *Origanum majorana* (F); a basal diet supplemented with 1 kg/ton *Salvia officinalis* and 1 kg/ton *Origanum majorana* (G). Feed conversion ratio (FCR).

**Table 4:** Effects of different treatments on egg quality in laying hens at 45 and 57 weeks of age.

Item	Treatments Group							SEM	*p*-Tukey
	A	B	C	D	E	F	G		
At 45 weeks of age									
Egg weight, g	56.06	55.88	56.03	56.06	56.02	56.01	56.02	0.63	0.932
Yolk color score	7.50	7.50	7.33	7.66	7.66	7.66	7.66	0.24	0.928
Shell thickness, µm	431.67	433.33	435.00	436.67	435.00	436.67	436.67	8.54	0.929
Yolk width, cm	3.73	3.62	3.45	3.48	3.47	3.60	3.60	0.10	0.386
Egg white width, cm	9.20	9.28	9.26	9.28	9.25	9.23	9.30	0.46	0.943
Yolk height, mm	16.34	16.35	16.24	16.24	16.29	16.29	16.22	0.40	0.944
Egg white height, mm	4.64	4.57	4.69	4.64	4.67	4.57	4.70	0.37	0.896
Yolk weight, g	16.43	16.17	16.63	16.43	16.60	16.60	16.61	0.48	0.798
Egg white weight, g	29.10	29.10	28.93	28.93	28.87	28.93	28.94	0.82	0.768
Shell weight, g	10.17	10.16	10.19	10.19	10.28	10.26	10.25	0.51	0.589
At 57 weeks of age									
Egg weight, g	64.84	64.98	65.22	64.95	64.9	64.26	64.8	0.33	0.485
Yolk color score	7.67 ^b^	8.33 ^ab^	8.83 ^a^	8.66 ^ab^	8.83 ^a^	7.67 ^b^	9.00 ^a^	0.24	0.007
Shell thickness, µm	426.67 ^b^	450.00 ^ab^	456.67 ^ab^	455.67 ^ab^	456.33 ^ab^	452.00 ^ab^	485.00 ^a^	10.43	0.031
Yolk width, cm	4.00	4.07	4.07	3.97	3.98	3.91	4.04	9.08	0.795
White width, cm	8.80 ^ab^	9.41 ^ab^	7.99 ^b^	7.72^b^	9.25 ^ab^	10.23^a^	9.27 ^ab^	0.46	0.008
Yolk height, mm	16.82	17.07	17.15	17.01	17.24	17.46	17.99	0.28	0.129
Egg white height, mm	5.25	5.94	5.88	5.95	5.94	6.01	6.01	0.35	0.741
Yolk weight, g	19.8	19.78	19.4	19.88	19.13	19.5	19.35	0.56	0.441
Egg white weight, g	33.17	33.15	33.17	33.19	33.67	32.45	33.19	1.16	0.671
Shell weight, g	11.58	12.02	12.37	11.59	11.82	12.03	12.05	0.81	0.793

^a,b^ The mean values followed by the different letters in the same row are significantly different (*p* < 0.05). The values presented are means and their standard error of 60 per treatment. Abbreviations: control (A); a basal diet supplemented with 0.5 *Salvia officinalis* (B); a basal diet supplemented with 1 kg/ton *Salvia officinalis* (C); a basal diet supplemented with 0.5 *Origanum majorana* (D); a basal diet supplemented with 1 kg/ton *Origanum majorana* (E); a basal diet supplemented with 0.5 kg/ton *Salvia officinalis* and 0.5 kg/ton *Origanum majorana* (F); a basal diet supplemented with 1 kg/ton *Salvia officinalis* and 1 kg/ton *Origanum majorana* (G).

**Table 5:** Effects of different treatments on internal organ weight and reproductive morphology of laying hens at 57 weeks.

Item	Treatments Group							SEM	*p*-Tukey
	A	B	C	D	E	F	G		
Live body weight, g	2007.5	2008.3	1998.3	2005.8	1990.8	1981.7	1982.5	27.5	0.426
Liver weight, g/100 g BW	2.75	2.78	2.85	2.74	2.68	2.80	2.87	0.29	0.219
Gizzard weight, g/100 g BW	1.01	0.91	1.00	1.04	1.04	1.07	1.09	0.081	0.130
Spleen weight, g/100 g BW	0.07	0.09	0.08	0.07	0.06	0.06	0.06	0.002	0.328
Heart weight, g/100 g BW	0.46	0.45	0.41	0.43	0.44	0.44	0.45	0.052	0.675
Abdominal fat, g/100 g BW	5.23	4.73	5.05	5.41	4.71	4.77	4.51	0.087	0.596
Ovary weight, g/100 g BW	2.65	3.03	2.68	2.68	2.78	3.51	3.16	0.26	0.154
Oviduct weight, g/100 g BW	3.23	3.70	3.65	3.56	3.73	3.51	3.56	0.19	0.112
Vagina weight, g/100 g OW	9.97	10.04	10.04	9.62	9.91	9.02	10.08	0.89	0.369
Uterus weight, g/100 g OW	29.24	29.48	31.30	30.75	31.11	32.06	31.68	1.42	0.336
Isthmus weight, g/100 g OW	14.92	14.32	15.14	14.02	15.03	15.01	14.02	1.22	0.211
Magnum weight, g/100 g OW	39.92	41.01	38.20	40.10	39.06	39.02	39.18	2.09	0.326
Infundibulum weight, g/100 g OW	5.95	5.15	5.32	5.51	4.89	4.89	5.03	0.43	0.221
Oviduct length, cm	55.03 ^b^	57.51 ^b^	57.61 ^ab^	58.00 ^a^	59.61 ^a^	58.87 ^a^	59.11 ^a^	3.10	0.025
Vagina length, cm	5.23	5.17	5.45	5.47	5.45	5.43	5.57	0.31	0.246
Uterus length, cm	6.07 ^c^	6.67 ^b^	6.50 ^b^	7.50 ^ab^	7.83 ^a^	7.95^a^	8.87 ^a^	0.27	0.015
Isthmus length, cm	10.33	10.17	10.83	10.17	10.67	10.83	10.17	0.93	0.199
Magnum length, cm	24.33	25.67	25.50	25.83	25.83	24.83	25.50	1.25	0.175
Inf. length, cm	9.07	9.83	9.33	9.03	9.83	9.83	9.00	0.34	0.148
LYF (>10 mm)	4.50 ^b^	5.33 ^a^	5.00 ^ab^	4.67 ^ab^	4.83 ^ab^	4.83 ^ab^	5.17^ab^	0.18	0.035
SYF (5 to 10)	11.83	12.00	12.67	12.00	11.83	11.50	10.50	0.59	0.298
LWF (3 to 5)	13.67 ^b^	18.50 ^ab^	20.33 ^a^	17.67 ^ab^	17.17 ^ab^	18.50 ^ab^	16.17^b^	1.41	0.063
MWF (1 to 3)	37.00 ^b^	50.33 ^a^	18.50 ^ab^	51.00 ^a^	48.83 ^a^	50.67 ^a^	46.17^ab^	2.48	0.004

^a,b,c^ The mean values followed by the different letters in the same row are significantly different (*p* < 0.05). The values presented are means and their standard error of 70 per treatment. Abbreviations: control (A); a basal diet supplemented with 0.5 *Salvia officinalis* (B); a basal diet supplemented with 1 kg/ton *Salvia officinalis* (C); basal diet supplemented with 0.5 *Origanum majorana* (D); a basal diet supplemented with 1 kg/ton *Origanum majorana* (E); a basal diet supplemented with 0.5 kg/ton *Salvia officinalis* and 0.5 kg/ton *Origanum majorana* (F); a basal diet supplemented with 1 kg/ton *Salvia officinalis* and 1 kg/ton *Origanum majorana* (G). large yellow follicle (LYF); small yellow follicle (SYF); large white follicle (LWF); medium white follicle (MWF); bodyweight (BW); oviduct weight (OW).

**Table 6:** Effects of different treatments on blood constituents of laying hens at 57 weeks of age.

Item	Treatments Group							SEM	*p*-Tukey
	A	B	C	D	E	F	G		
Glucose, mg/dl	167.67 ^a^	134.33 ^ab^	130.67 ^b^	135.00 ^ab^	143.00 ^ab^	141.33 ^ab^	128.20 ^b^	9.19	0.034
GPT, IU/L	24.83	24.97	25.16	24.87	24.87	24.90	24.87	0.86	0.437
GOT, IU/L	122.50	120.67	122.67	125.00	125.00	128.33	123.33	10.17	0.536
Total protein, mg/dL	5.37	5.37	5.50	6.30	5.80	6.07	6.13	0.33	0.322
Albumin, mg/dL	1.63	1.60	1.70	1.70	1.90	1.73	1.77	0.09	0.351
Globulin, mg/dL	3.73	3.77	3.80	4.60	3.90	3.90	4.10	0.37	0.625
Cholesterol, mg/dL	150.67 ^a^	147.33 ^a^	149.67 ^a^	128.33 ^c^	130.17 ^b^	118.33 ^c^	118.53 ^c^	13.29	0.001
Triglyceride, mg/dL	172.00 ^a^	168.67 ^a^	165.33 ^a^	148.33 ^b^	151.33 ^b^	132.33 ^c^	140.00 ^bc^	12.71	0.001
HDL, mg/dL	50.33	61.33	61.33	58.67	59.00	60.67	61.00	6.02	0.195
LDL, mg/dL	93.27 ^a^	87.27 ^ab^	88.87 ^ab^	70.67 ^b^	72.07 ^bc^	58.87 ^c^	59.67 ^c^	6.95	0.008

^a,b,c^ The mean values followed by the different letters in the same row are significantly different (*p* < 0.05). The values presented are means and their standard error of 60 per treatment. Abbreviations: control (A); a basal diet supplemented with 0.5 *Salvia officinalis* (B); a basal diet supplemented with 1 kg/ton *Salvia officinalis* (C); basal diet supplemented with 0.5 *Origanum majorana* (D); a basal diet supplemented with 1 kg/ton *Origanum majorana* ©; a basal diet supplemented with 0.5 kg/ton *Salvia officinalis* and 0.5 kg/ton *Origanum majorana* (F); a basal diet supplemented with 1 kg/ton *Salvia officinalis* and 1 kg/ton *Origanum majorana* (G).

**Table 7:** Effect of the different treatments on fatty acids ratio in the yolk of laying hens at 57 weeks.

Item	Treatments Group							SEM	*p*-Tukey
	A	B	C	D	E	F	G		
Myristic acid (C14:0)	0.221	0.223	0.224	0.226	0.212	0.226	0.221	0.022	0.54
Palmitic acid (C16:0)	25.827	24.993	25.452	25.410	25.748	25.979	25.917	1.733	0.36
Palmitoleic acid (C16:1)	3.888 ^a^	2.765 ^ab^	2.436 ^b^	2.342 ^b^	2.389 ^b^	2.376 ^b^	2.291 ^b^	0.633	0.043
Stearic acid (C18:0)	9.783 ^a^	7.977 ^b^	7.823 ^b^	7.970 ^b^	7.789 ^b^	7.972 ^b^	7.624 ^b^	0.839	0.037
Oleic acid (C18:1 n-9c)	42.296	43.982	43.543	43.879	43.739	43.213	43.645	3.854	0.322
Vaccenic acid (C18:1 n-7)	1.954	1.896	1.943	1.978	1.959	1.879	1.922	0.213	0.413
Linoleic acid (C18:2 n-6)	12.428 ^b^	14.638 ^a^	14.956 ^a^	14.637 ^a^	14.557 ^ab^	14.701 ^a^	14.684 ^a^	1.023	0.048
α-linolenic acid (ALA, C18:3 n-3)	0.528	0.537	0.557	0.558	0.569	0.565	0.569	0.031	0.084
Arachidonic acid (AA, C20:4 n-6)	1.889	1.817	1.823	1.819	1.826	1.911	1.921	0.085	0.124
Eicosapentaenoic acid (EPA, C20:5 n-3)	0.087	ND	0.087	ND	0.086	ND	0.088	0.0001	0.824
Docosapentaenoic acid (DPA, C22:5 n-3)	0.117	0.118	0.118	0.117	0.117	0.119	0.119	0.001	0.242
Docosahexaenoic acid (DHA, C22:6 n-3)	0.963	0.965	0.967	0.968	0.967	0.983	0.962	0.052	0.345

^a,b^ The mean values followed by the different letters in the same row are significantly different (*p* < 0.05). The values presented are means and their standard error of 60 per treatment. Abbreviations: control (A); a basal diet supplemented with 0.5 *Salvia officinalis* (B); a basal diet supplemented with 1 kg/ton *Salvia officinalis* (C); basal diet supplemented with 0.5 *Origanum majorana* (D); a basal diet supplemented with 1 kg/ton *Origanum majorana* (E); a basal diet supplemented with 0.5 kg/ton *Salvia officinalis* and 0.5 kg/ton *Origanum majorana* (F); a basal diet supplemented with 1 kg/ton *Salvia officinalis* and 1 kg/ton *Origanum majorana* (G). Not detected, (ND).

## Data Availability

Upon request from the corresponding author.
